# Supplementary material for: SPRTN is a mammalian DNA-binding metalloprotease that resolves DNA-protein crosslinks
Source: eLife. 2016 Nov 17;5:e21491. doi: 10.7554/eLife.21491 (PMC5127644; doi:10.7554/eLife.21491)
Supplement: Supplemental file 1. — DOI: http://dx.doi.org/10.7554/eLife.21491.014 [file elife-21491-fig1.docx]

| **Strain Name** | **Mating type** | **Genotype** | **Source** |
| --- | --- | --- | --- |
| yJLO0 | Mat a | [BMA64-1A] *leu2-3,112 his3-11,15 trp1∆ ade2-1 ura3-1 can1-100* | Euroscarf |
| yJLO1 | Mat a | *tdp1∆::HpHMX* | This study |
| yJLO2 | Mat a | *wss1∆::TRP1 tdp1∆::HpHMX* | This study |
| yJLO3 | Mat a | *wss1∆::TRP1 tdp1∆::HpHMX ura3-1::pGAL1-10 FLAG-SPRTN::URA3* | This study |
| yJLO4 | Mat a | *wss1∆::TRP1 tdp1∆::HpHMX ura3-1::pGAL1-10 FLAG-sprtn-E112A::URA3* | This study |
| yKM8 | Mat a | *[BY4741]his3-11,15, leu2-3,112, trp1-1, ura3-1, ade2-1, can1-100, lys2::tTA, ura3::pCMVtetR'-SSN6 KlURA3* | Gnanasundram, S.V. et al. |
| yKM9 | Mat a | *tdp1∆::HpHMX* | This study |
| yKM10 | Mat a | *tdp1∆::HpHMX PtetO7-Ubi-3xHA-Wss1-NatMX4* | This study |
| yKM48 | Mat a | *tdp1∆::HpHMX PtetO7-Ubi-3xHA-Wss1-NatMX4 [pRS415]* | This study |
| yKM49 | Mat a | *tdp1∆::HpHMX PtetO7-Ubi-3xHA-Wss1-Nat-MX4 [pWSS1-GFP-SPRTN-pRS415]* | This study |
| yKM50 | Mat a | *tdp1∆::HpHMX PtetO7-Ubi-3xHA-Wss1-NatMX4 [pWSS1-GFP-sprtn-E112A-pRS415]* | This study |
| yKM51 | Mat a | *tdp1∆::HpHMX PtetO7-Ubi-3xHA-Wss1-NatMX4 [pWSS1-GFP-sprtn-Y117C-pRS415]* | This study |
| yKM52 | Mat a | *tdp1∆::HpHMX PtetO7-Ubi-3xHA-Wss1-NatMX4 [pWSS1-GFP-sprtn-∆C-pRS415]* | This study |

**Supplemental file 1**

yJLO3 was derived from yJLO2; yJLO2 was derived from yJLO1; yJLO1 was derived from yJLO0. yKM9 was derived from yKM8. yKM10, yKM48, yKM49, yKM50, yKM51 and yKM50 were derived from yKM9.
